# Active immunisation targeting nerve growth factor attenuates chronic pain behaviour in murine osteoarthritis

**DOI:** 10.1136/annrheumdis-2018-214489

**Published:** 2019-03-12

**Authors:** Isabell S von Loga, Aadil El-Turabi, Luke Jostins, Jadwiga Miotla-Zarebska, Jennifer Mackay-Alderson, Andris Zeltins, Ida Parisi, Martin F Bachmann, Tonia L Vincent

**Affiliations:** 1 Kennedy Institute of Rheumatology, University of Oxford, Oxford, UK; 2 The Jenner Institute, University of Oxford Medical Sciences Division, Oxford, UK; 3 Molecular Microbiology and Virology, Latvian Biomedical Research & Study Centre, Riga, Latvia; 4 RIA, Immunology, Inselspital, 3010 Bern, Switzerland

**Keywords:** immunization, vaccine, osteoarthritis, chronic pain, nerve growth factor

## Abstract

**Objectives:**

Nerve growth factor (NGF) has emerged as a key driver of pain in osteoarthritis (OA) and antibodies to NGF are potent analgesics in human disease. Here, we validate a novel vaccine strategy to generate anti-NGF antibodies for reversal of pain behaviour in a surgical model of OA.

**Methods:**

Virus-like particles were derived from the cucumber mosaic virus (CuMV) and coupled to expressed recombinant NGF to create the vaccine. 10-week-old male mice underwent partial meniscectomy to induce OA or sham-surgery. Spontaneous pain behaviour was measured by Linton incapacitance and OA severity was quantified using OARSI histological scoring. Mice (experimental and a sentinel cohort) were inoculated with CuMVtt^NGF^ (Vax) or CuMVtt^ctrl^ (Mock) either before surgery or once pain was established. Efficacy of anti-NGF from the plasma of sentinel vaccinated mice was measured in vitro using a neurite outgrowth assay in PC12 cells.

**Results:**

Anti-NGF titres were readily detectable in the vaccinated but not mock vaccinated mice. Regular boosting with fresh vaccine was required to maintain anti-NGF titres as measured in the sentinel cohort. Both prophylactic and therapeutic vaccination demonstrated a reversal of pain behaviour by incapacitance testing, and a meta-analysis of the two studies showing analgesia at peak anti-NGF titres was highly statistically significant. Serum anti-NGF was able to inhibit neurite outgrowth equivalent to around 150 ug/mL of recombinant monoclonal antibody.

**Conclusions:**

This study demonstrates therapeutic efficacy of a novel NGF vaccine strategy that reversibly alleviates spontaneous pain behaviour in surgically induced murine OA.

**Key messages**

**What is already known about this subject?**

- Nerve growth factor (NGF) is a validated target for pain in human and mouse OA.
- Neutralising antibodies to NGF show therapeutic efficacy in Phase III clinical studies.

**What does this study add?**

- Here, we demonstrate efficacy of an NGF vaccine that reversibly induces neutralising anti-NGF antibodies and suppresses pain behaviour in murine OA.

**How might this impact on clinical practice or future developments?**

- Vaccination potentially offers a tuneable, cheaper and easier to use alternative to biological therapy in patients.

OA is the most prevalent joint disease costing approximately 1%–2.5% of the gross domestic product of developed countries.^1^ Greater than 75% of patients experience pain on a daily basis.^2^ Current standard therapies for pain relief, such as non-steroidal anti-inflammatory drugs (NSAIDs) and opioids are limited by their modest efficacy and long-term safety.^3^ In the last decade, nerve growth factor (NGF), a key pain sensitiser, has emerged as a promising target for OA pain. In humans, neutralising antibodies to NGF significantly suppress pain associated with late-stage OA.^4^ However, biological therapy in OA is likely to be limited by cost^5^ and by treatment failure due to anti-drug antibodies.^6^ Active immunisation targeting NGF represents an attractive alternative to deliver effective analgesia, while potentially providing a more economically sustainable substitute for patients. The latter is particularly the case as biosimilars replace proprietary products.^7^

Chronic pain in late OA can be modelled using surgical models of joint destabilisation in mice. Spontaneous pain behaviour is assessed by differential weight distribution of the hind limbs using incapacitance testing. Following joint destabilisation, mice display two phases of pain behaviour: one immediately following surgery (postoperative pain) and a second late phase that starts between weeks 7 and 11 after surgery and which is progressive (online supplementary figure 1a) with worsening joint destruction (online supplementary figure 1b, c).^8 9^ Both phases of pain behaviour correspond to an increase in NGF expression in the joint (online supplementary figure 1d, e)^10 11^ and can be neutralised by delivery of NGF’s soluble receptor (TrkAd5).^10^

10.1136/annrheumdis-2018-214489.supp1 Supplementary data

Immunisation against self-proteins can be achieved by displaying the antigen of interest on virus-like particles (VLPs). Due to their optimal size and geometry, VLPs can effectively transit to draining lymph nodes to drive antigen-dependent immunogenicity.^11^ Antigens are arranged as a repetitive array on the particles’ surfaces via genetic fusion or chemical conjugation to generate good polyclonal antibody responses without breaking T cell tolerance. This means that the antibody response will only occur when the antigen is presented on the VLP.^12 13^

Here, we describe a novel plant virus derived VLP based on the cucumber mosaic virus,^14^ that incorporates a tetanus toxoid epitope for T cell help (herein referred to as CuMVtt, figure 1A).^15 16^ Addition of a non-coding, 3' untranslated region in the VLP expression construct, leads to increased retention of encapsulated RNA suggesting greater particle stability (online supplementary figure 2a). Purified His-tagged NGF was covalently conjugated to CuMVtt (online supplementary figure 2b) as previously described for RNA-phage based VLPs.^17^ Native conformation of recombinant NGF was tested by its ability to bind a neutralising monoclonal antibody and the interacting domain of the high-affinity receptor (TrkA-d5) (online supplementary figure 2c, d).

10.1136/annrheumdis-2018-214489.supp2 Supplementary data

**Figure 1:**
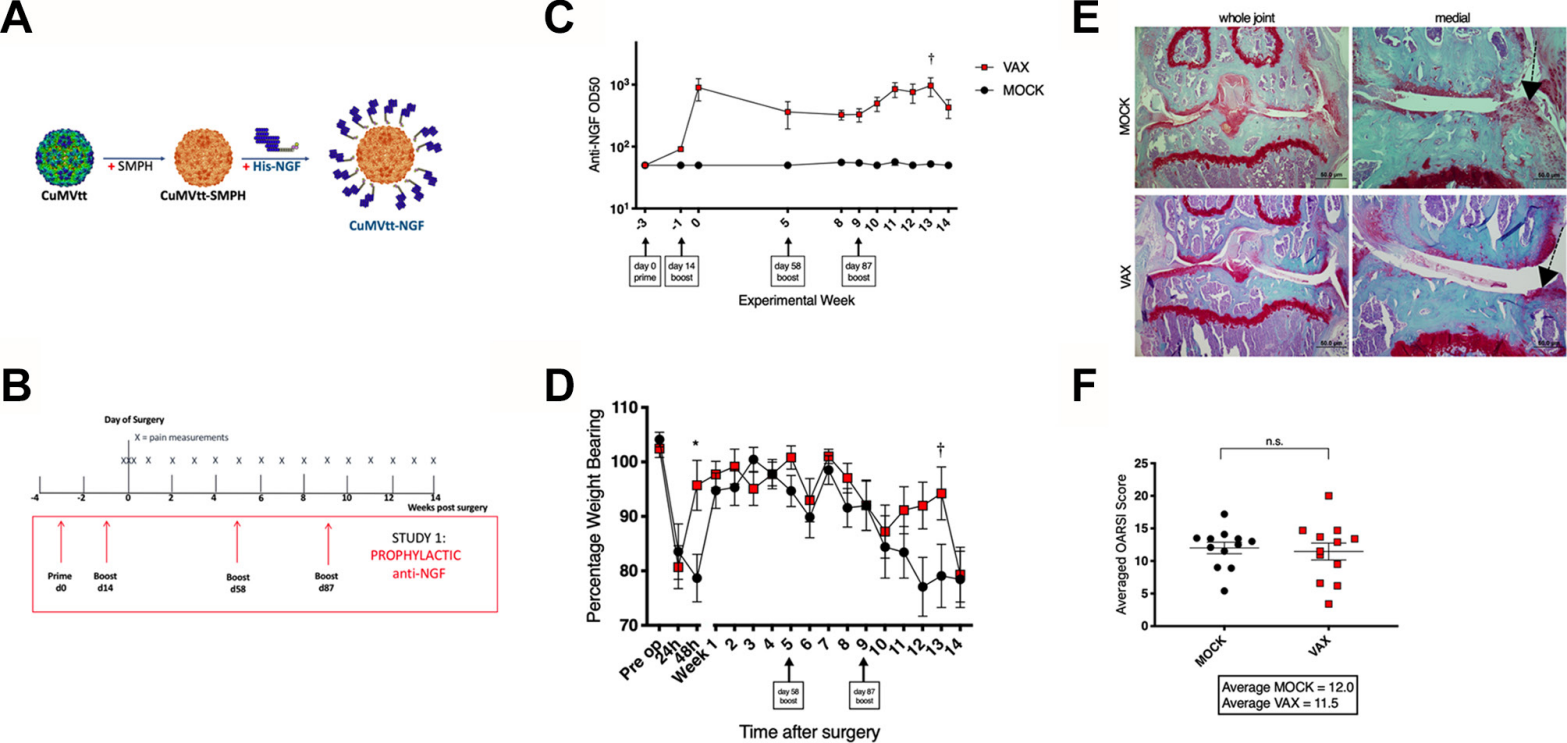
Prophylactic NGF vaccination blocks murine OA pain. (A) VLP is chemically cross-linked (SMPH) to enable conjugation with His-NGF. (B) Prophylactic vaccination protocol. (C) Anti-NGF titres in sentinel cohort (n=10). (D) Painful behaviour following surgical induction of OA (n=40) measured by Linton incapacitance where 100% represents equal weight distributed across R and L limbs. Repeated-measures two-way ANOVA with Bonferroni multiple comparisons test applied, *adjusted p<0.05. SEM shown. Differences between treatment groups during late OA pain phase were not significant after correcting for multiple testing. † identifies time point of highest anti-NGF titre (see figure 2D). (E) Representative histological sections for (F) cartilage degradation (OARSI) scores 18 weeks after PMX surgery in mice treated with mock or NGF vaccine. Statistical significance is shown by two-tailed t-test. Bars represent mean±SEM, n.s.—non-significant., **p<0.01 by t-test. CuMVtt adapted from EMD: 3855.^14^ ANOVA, analysis of variance; NGF, nerve growth factor; PMX, partial meniscectomy; VLP, virus-like particle.

To test the therapeutic efficacy of NGF vaccination, mice were immunised with either CuMVtt^NGF^ (Vax) or CuMVtt^ctrl^ (Mock) 2 weeks prior to joint destabilisation (figure 1B). Non-operated sentinel control mice also underwent vaccination to enable regular blood sampling over the experimental course. Immunisation led to seroconversion by week 3, followed by a decline in antibody titres. Additional boosts were necessary to maintain antibody levels (figure 1C). No difference in pain behaviour was detected in NGF immunised animals 24 hours postoperatively (postop), but CuMVtt^NGF^ vaccinated animals recovered from pain behaviour faster than mock-vaccinated animals (within 48 hours) (figure 1D). As expected mice were pain free for several weeks, but pain behaviour started to develop from 8 weeks postsurgery. Following a boost at 10 weeks postop, and in keeping with a concomitant rise in the serum levels of anti-NGF antibody, a reversal of pain behaviour was observed. This was maintained for 3 weeks until anti-NGF titres fell and pain behaviour resumed. At termination of the experiment, joints were harvested and scored for OA severity. No difference in disease severity between mock and vaccinated groups was observed (figure 1E,F). Sera were also collected from experimental mice at the end of the study (week 18) to measure general antibody responses. Anti-CuMV IgG levels were elevated in both vaccinated and mock-vaccinated groups compared with non-vaccinated control animals. Total IgG and IgM levels were largely consistent across all groups. There was no evidence of induction of autoantibodies such as rheumatoid factor in any of the groups (online supplementary figure 3).

10.1136/annrheumdis-2018-214489.supp3 Supplementary data

A second experiment was carried out to establish whether analgesia could be induced by immunisation after induction of pain behaviour i.e. therapeutic vaccination (figure 2A). When pain behaviour was established (10 weeks postop) mice were randomised into two groups: vaccinated and mock-vaccinated. Vaccine boosts were delivered at weeks 12 and 15 postop to maintain titres. Higher titre anti-NGF levels at the end of the experiment (around OD50 10^3^) appeared to be associated with an analgesic response between weeks 14 and 18 postop (figure 2B,C). A subsequent spontaneous reduction in titres was associated with resumption of pain behaviour. Direct correlation between antibody levels and pain behaviour during the experiment was not possible as titres were only measured in the sentinel and not the experimental group. A meta-analysis comparing the analgesic effects across both studies at the point of highest titre in the sentinel group (week 13 for the prophylactic study and week 17 for the therapeutic study, marked by †) yielded a significant difference (p=8.93e-05) between mock and vaccinated cohorts (figure 2D). No heterogeneity of effect was detected between the two studies (I^2^=0, p=0.827). The sentinel cohort was maintained to follow the fall in antibody titres over the following 10 weeks, which was similar to that observed in previous studies.^17^ IgG purified from the serum of CuMVtt^NGF^ vaccinated, but not control mice was able to dose-dependently inhibit NGF induced neurite outgrowth in PC-12 cells (figure 2E,F), to a level similar to that seen with 150 ug/mL monoclonal anti-NGF antibody (online supplementary figure 4). The effect appeared to plateau after 5 ug/mL.

10.1136/annrheumdis-2018-214489.supp4 Supplementary data

**Figure 2:**
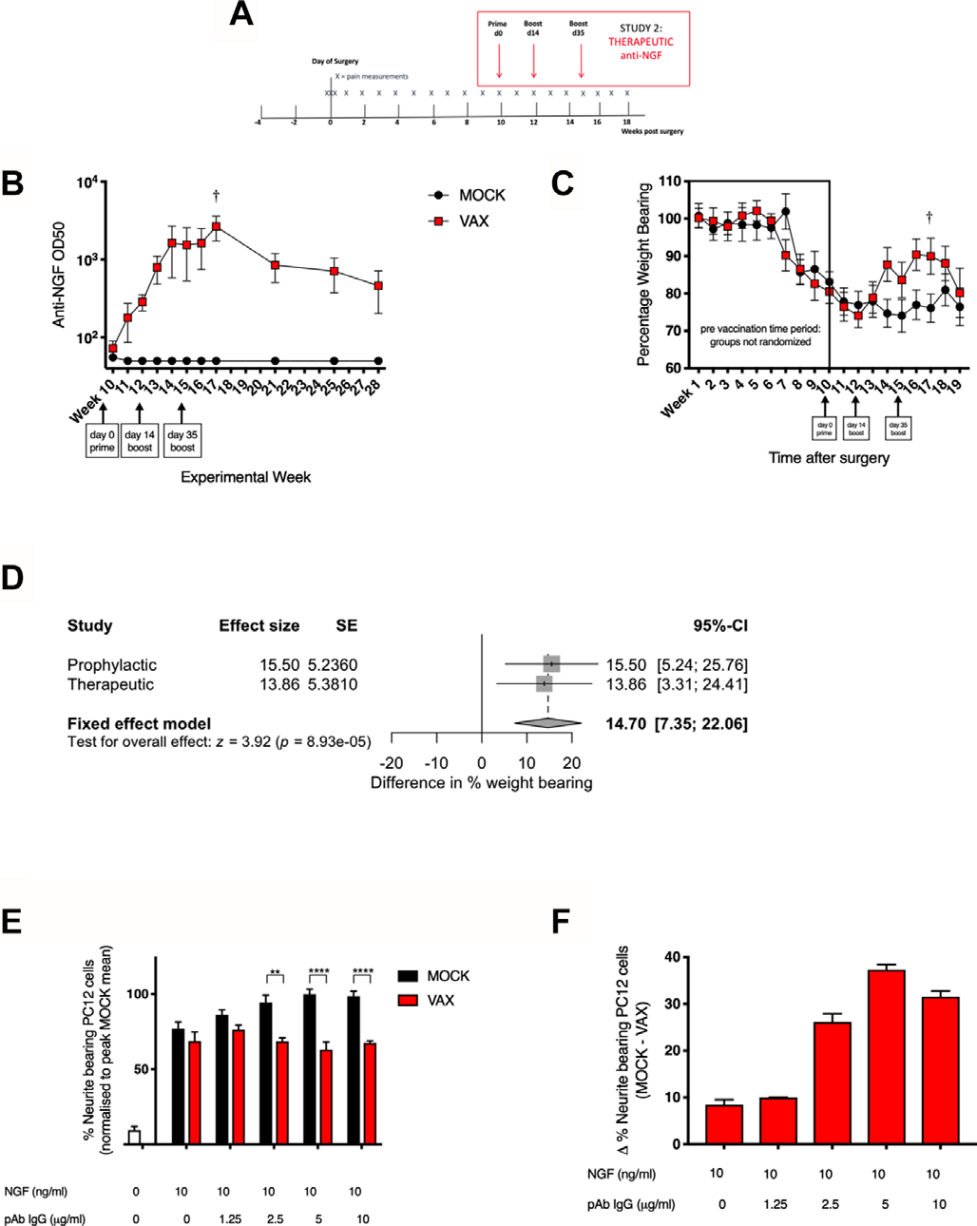
Therapeutic NGF vaccination reduces murine OA pain. (A) Therapeutic vaccination protocol. (B) Anti-NGF titres in sentinel cohort (n=10). (C) Painful behaviour measured by incapacitance testing where 100% represents equal weight distributed across R and L Limbs (n=40). Mice were randomised to receive mock or NGF vaccine at 10 weeks postsurgery. Repeated-measures two-way ANOVA with Bonferroni multiple comparisons test applied. SEM shown. Differences between treatment groups during late OA pain phase were not significant after correcting for multiple testing (D) Forest plot of meta-analysis comparing the effect size of analgesic response between mock and vaccinated cohorts at points of highest titre in the sentinel groups (week 13 for the prophylactic study, week 17 for the therapeutic study, marked by †). (E) Neurite outgrowth inhibition with increasing concentrations of IgG isolated from serum of vaccinated animals and (F) their normalised difference compared with mock-vaccinated animals. Bars represent mean±SEM, *p<0.05, ***p<0.001, ****p<0.0001 by t-test. ANOVA, analysis of variance; NGF, nerve growth factor.

Vaccines to self-antigens have been developed for other non-communicable diseases over the years. Early studies showed preclinical success but with limited clinical efficacy, which may have been due to poor immunogenicity of the vaccine platform, requiring the use of codelivery of adjuvant in preclinical models. Recent studies using refined vaccine platforms have demonstrated translatable efficacy from mouse to large animals including humans.^18–20^ Our results show that CuMVtt^NGF^ vaccination produces analgesia in mice when delivered both before and after pain behaviour has become established. A unique aspect of this study is to combine a novel VLP-based therapeutic vaccine with measures of spontaneous pain behaviour in murine OA; its success confirming NGF as a valid target for OA related pain.^21^

Implementation of this type of strategy to treat OA pain has additional benefits. It induces a polyclonal response that might be more effective than a recombinant monoclonal antibody as it will stimulate antigen removal mediated by Fc-dependent clearance mechanisms.^7^ It should also prevent a reduction in efficacy over time by anti-idiotypic antibodies. However, safety is also a concern. Accelerated arthropathy (rapidly progressive OA, RPOA) has been described in a small proportion of patients receiving high dose anti-NGF therapy, especially in combination with NSAIDs.^3^ The mechanism for this is unclear and may be related to loss of joint protection when pain is abrogated or due to, as yet, undefined disease modifying actions of NGF.^3^ It is therefore reassuring that this vaccination strategy does not induce long-lived antibody responses and requires regular boosting to maintain titres. While we did not observe accelerated disease in our NGF-vaccinated cohort, we recognise that safety remains a significant issue, and this would need to be monitored carefully in any future clinical development. This proof of concept study has significant translational potential; in the first instance within veterinary practice where activity measures are validated pain outcomes.^22^ Ultimately, this has the potential to reduce the burden of disease in humans (online supplementary files 5–7).

10.1136/annrheumdis-2018-214489.supp5 Supplementary data

10.1136/annrheumdis-2018-214489.supp6 Supplementary data

10.1136/annrheumdis-2018-214489.supp7 Supplementary data
