# Supplementary figures and images for: Active immunisation targeting nerve growth factor attenuates chronic pain behaviour in murine osteoarthritis
Source: Ann Rheum Dis. 2019 Mar 12;78(5):672–5. doi: 10.1136/annrheumdis-2018-214489 (PMC6517802; doi:10.1136/annrheumdis-2018-214489)

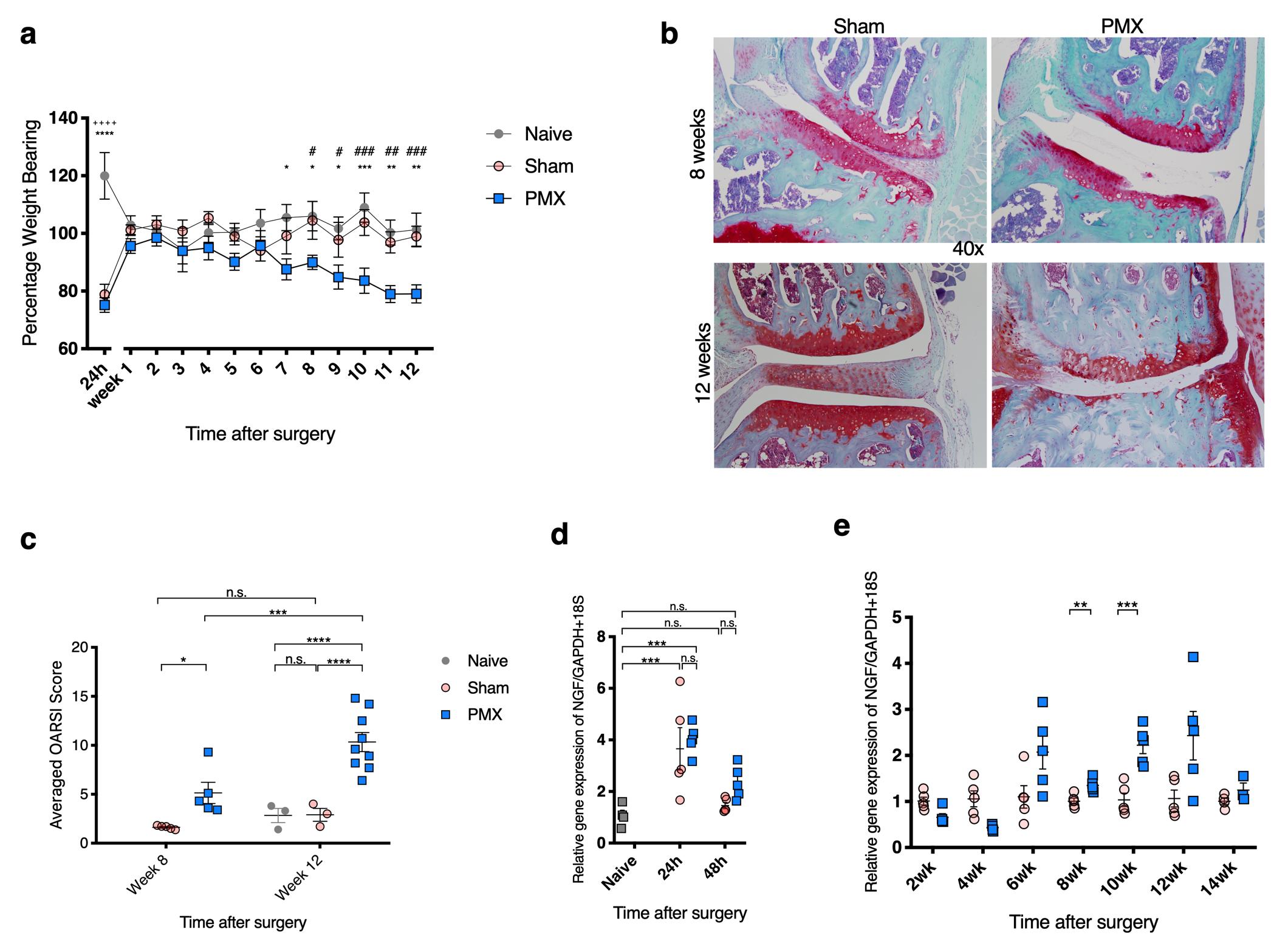

Supplement: Supplementary data [file annrheumdis-2018-214489supp001.jpeg]

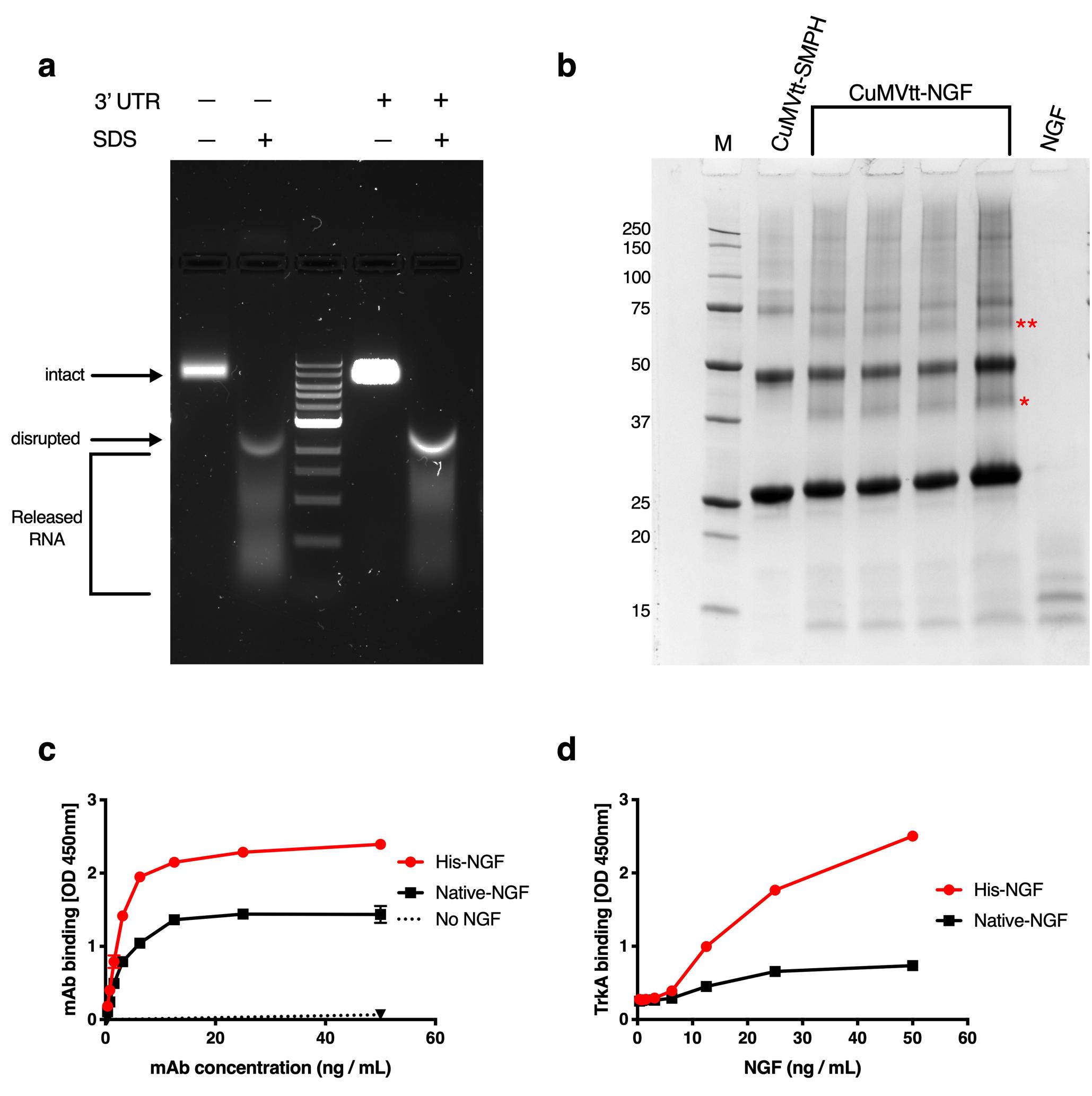

Supplement: Supplementary data [file annrheumdis-2018-214489supp002.jpeg]

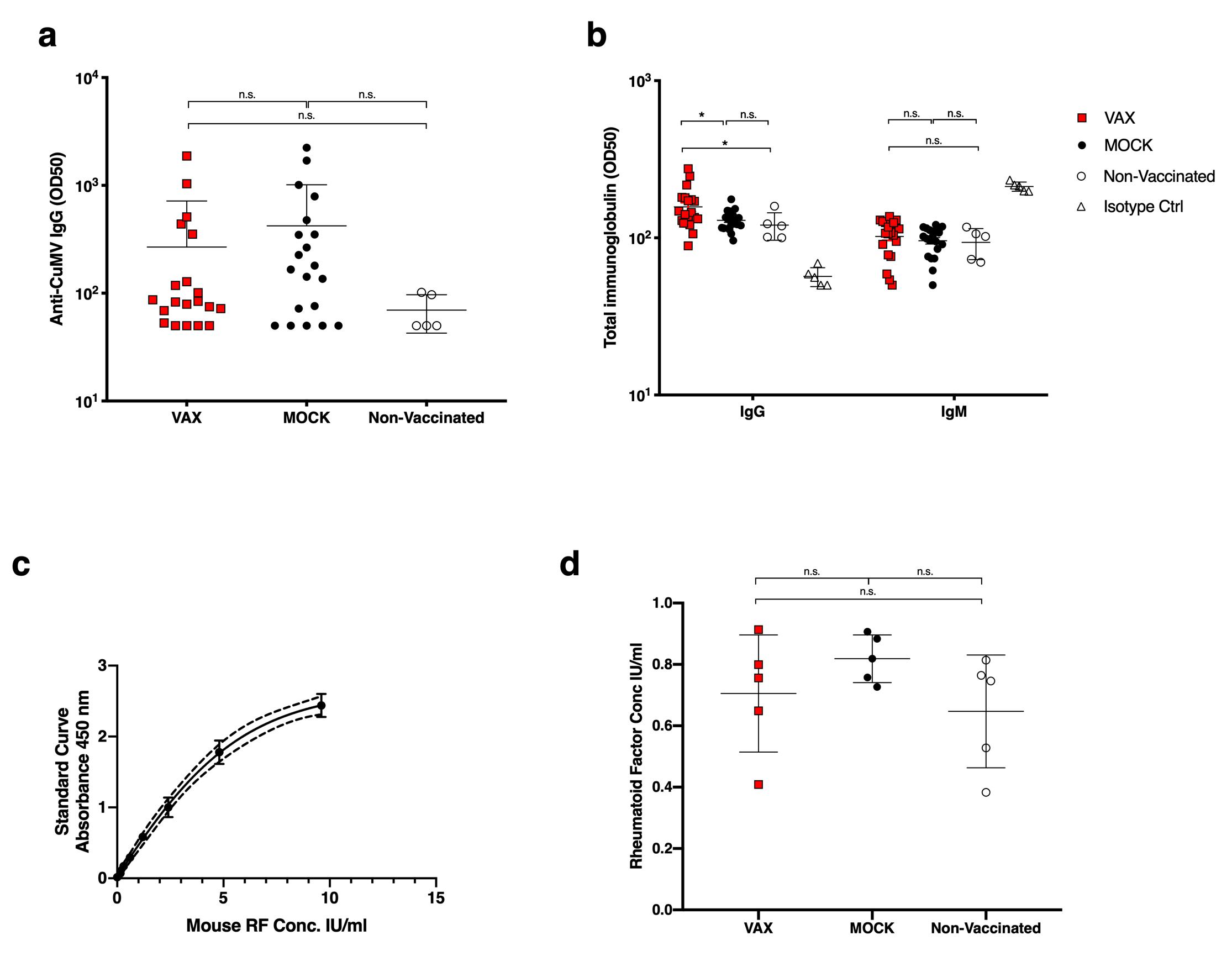

Supplement: Supplementary data [file annrheumdis-2018-214489supp003.jpeg]

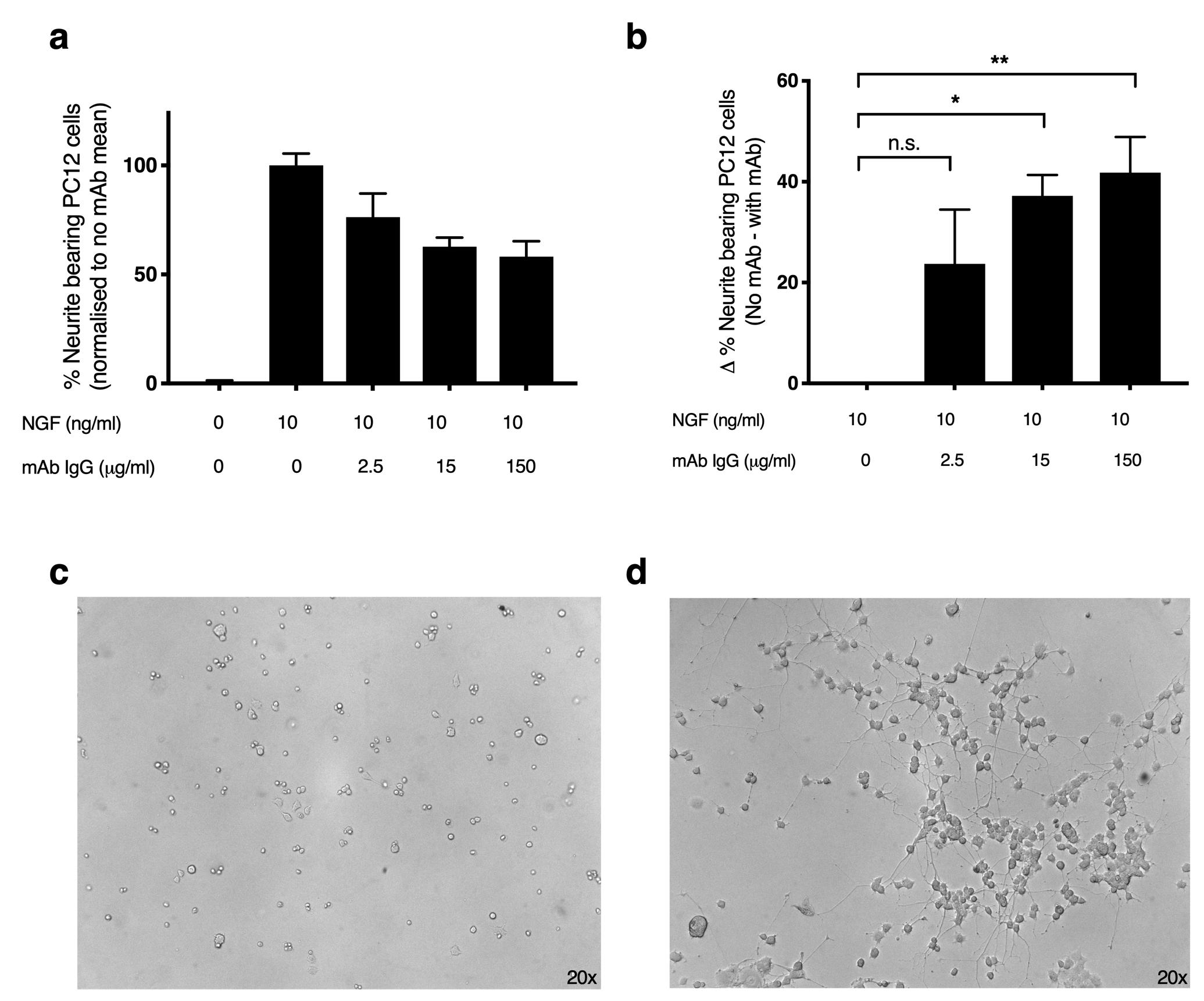

Supplement: Supplementary data [file annrheumdis-2018-214489supp004.jpeg]
