# Supplementary material for: Active immunisation targeting nerve growth factor attenuates chronic pain behaviour in murine osteoarthritis
Source: Ann Rheum Dis. 2019 Mar 12;78(5):672–5. doi: 10.1136/annrheumdis-2018-214489 (PMC6517802; doi:10.1136/annrheumdis-2018-214489)
Supplement: Supplementary data [file annrheumdis-2018-214489supp005.docx]

**Contributions**

A.E.T., I.v.L. and T.V. designed the studies. M.F.B. originated the concept of the vaccine. J.Z. and I.v.L. conducted the mouse surgery and I.v.L. conducted the behavioural studies. A.Z. provided VLP constructs and developed purification strategies. A.E.T. produced and characterised the CuMVtt^NGF^ vaccine. A.E.T. performed the vaccinations and immunological assays. I.P. performed the histological preparation and in conjunction with I.v.L. conducted the histological analysis. L.J. conducted and approved of the statistical analysis. J.A. conducted the ELISA experiment.

**Supplementary information**

Supplementary Figures: Figure S1 – Figure S4

Online Methods

Reporting Summary = details of statistical parameters, software and code, data availability, study design (sample size, data exclusion, replication, randomisation, blinding), Materials & experimental systems.
