# Supplementary material for: Active immunisation targeting nerve growth factor attenuates chronic pain behaviour in murine osteoarthritis
Source: Ann Rheum Dis. 2019 Mar 12;78(5):672–5. doi: 10.1136/annrheumdis-2018-214489 (PMC6517802; doi:10.1136/annrheumdis-2018-214489)
Supplement: Supplementary data [file annrheumdis-2018-214489supp006.docx]

**METHODS**

**Recombinant constructs and protein purification**

BL21(DE3)-Star E. *coli* (Thermofisher Scientific) were transformed with pET42-CMVNtt830-3UTR that contains a native non-coding 3’ untranslated region, and the expressed VLPs were prepared as previously described[14]. The NGF construct design was based on Röhn *et al*[17]*.*

An oligonucleotide of murine beta-NGF sequence (UniProt # P01139) corresponding to aa19-121 with additional immunoglobulin kappa chain signal sequence at the 5’ and further sequence encoding hexahistidine (6H) tag and a glycine-glycine-cysteine (GGC) at the 3’ end, flanked by restriction enzymes BamHI 5’ and XhoI 3’ was synthesised as a GeneString (Thermofisher Scientific). Restriction digest allowed directional cloning in to the mammalian expression vector, pHLsec[23], creating pHLsec-betaNGF. PEI transfected HEK293T cells were transferred to expanded surface roller bottles and recombinant NGF purified from culture supernatants (OptiMEM, L-Glutamine, 2% FBS, NEA, Pen/ Strep, acetyl cysteine, reduced glutathione) after 5 days. Culture supernatant volumes were concentrated by tangential flow filtration (AKTA Flux S, GE Healthcare) with 10,000 MWCO flat sheet membrane cassette (GE Healthcare) ahead of Ni^2+^ affinity chromatography (NAC) recirculating sample (12 – 16 h) at 4^o^C (HisTrap Excel nickel affinity column, GE Healthcare). Bound NGF was eluted by imidazole elution (50mM Na-Phosphate, pH8, 150mM NaCl, 0.5 M imidazole, 10mM B-mercaptoethanol buffer) and concentrated with Amicon Ultra centrifugal filters (3,000 MWCO, Millipore) ahead of size exclusion chromatography (SEC) (Sephadex 75 16/600 column, GE Healthcare) with isocratic elution in 20mM MES pH 6.0, 10% glycerol. NGF positive fractions, as determined by ~14kDa band in SDS-PAGE and anti-6H western blot, were pooled and the protein concentration adjusted to 0.3 – 0.6 mg/ ml. Recombinant NGF was aliquoted and stored frozen or kept on ice for immediate further use.

**Vaccine production and immunisation.**

CuMVtt was derivatised with 7.5 x molar excess SMPH (30 min, 22^o^C) and diafiltered in to coupling buffer (20 mM MES pH 6.0, 30% sucrose (w/v)) with 100,000 MWCO Amicon Ultra centrifugal filters (Millipore). The CuMVtt-SMPH was immediately mixed with NGF (pre-treated for 1 h with 10 x molar excess TCEP) and incubated for 4 - 8 h at 5^o^C with continuous gentle agitation 400rpm (Eppendorf thermomixer C, Eppendorf) to produce CuMVtt^NGF^. Vaccine suspension had a lightly turbid appearance (no large aggregates visible) and was immediately aliquoted, sufficient for 20 x 50 μg doses and stored frozen at -20^o^C. Aliquots of CuMVtt alone diafiltered into MES buffer were likewise aliquoted and stored.

Immunisation was performed on restrained mice. 50 μg in 100 μl volume was applied slowly via 30G BD Micro-Fine+ syringe (Becton Dickinson) subcutaneously to the dorsal region. Routine small blood samples were collected by needle puncture of tail lateral vein. Terminal blood samples were collected by cardiac puncture.

Antibody titres were measured by indirect ELISA. 50 µl recombinant NGF (prepared in-house), diluted in carbonate bicarbonate buffer (CBB) (2 µg/ ml), were coated on 96-well microtitre plate (Nunc immunosorb) incubating 12 – 16 h at 4^o^C. Plates were blocked for 2 h at room temperature (21-25^o^C) with blocking solution (2% BSA in PBS- 0.05% Tween 20 (PBS-T)). Serum samples from individual animals were diluted 1:50 with blocking solution, and 150 µl was added to well. From there 50 µl was transferred to 100 µl blocking solution in the next well to produce a 1:3 dilution and this was repeated across the remaining wells. Samples were incubated at room temperature for 2 h, and plates were washed four times with PBS-T. 100 µl of anti-mouse IgG HRP-conjugate (Sigma) 1:5000 in PBS-T was then added to each well and incubated for 1 h at room temperature, then washed four times as before. Bound conjugate signal was developed for up to 5 min at room temperature with 100 µl of 3,3',5,5'-tetramethylbenzidine (TMB) substrate, and stopped by the addition of 100 µl of stop solution (0.16M H_2_SO_4_). Plates were read, measuring absorbance at 450 nm on a CLARIOstar microplate reader (BMG Labtech) using MARS software (version 3.10 R5). Total immunoglobulin was detected by indirect ELISA, coating plates overnight with sera from individual PMX-operated mice 18 weeks after mock or NGF vaccination. A non-vaccinated group was used as a control. Serum samples were diluted 1:100 in CBB prior to coating the plates. Control wells were coated with mouse IgG1 mAb (Alomone) or mouse IgM (eBioscience) diluted to 1ug/ml in CBB as isotype controls. Bound immunoglobulins were probed with isotype-specific rabbit anti-mouse antibodies against IgG lambda or IgM (Bio-rad) (1:3 serial dilution, starting at 1:1000). After washing (PBS-T), bound Ig was detected with goat anti-rabbit-HRP conjugate (1:3000 dilution), visualised with TMB as before. Rheumatoid factor (RF) assay was from Bioassay Technology Laboratory, Shanghai (cat no. E1152Mo) and performed on serum samples diluted 1:10 following manufacturer’s instructions.

**NGF in vitro bioactivity assays**

The native conformation of recombinant His-NGF was tested by recognition with a neutralising mAb (MAB256, R&D systems, UK) or binding the interacting domain of the high affinity receptor (TrkAd5-Fc chimera, R&D systems, UK). Microtitre plates were coated with 50 µl His-NGF in CBB (fixed 0.05 µg NGF/ well for mAb, and 1:2 dilution starting at 0.05 µg NGF/ well for TrkAd5-Fc) and processed as for indirect ELISA (see above). Following incubation (2h) with 200 µl blocking solution, the plates were washed and incubated with a 1:2 dilution series, starting at 0.05 µg/ml (100 µl), of MAB256 or 0.1 µg/ml (100 µl) TrkAd5-Fc (in triplicate). Wells were washed thoroughly with PBS-T, bound MAB256 was detected with (100 µl) anti-mouse IgG HRP-conjugate (Sigma) 1:5000 in PBS-T, whereas bound TrkAd5-Fc was detected with (100 µl) anti-human IgG HRP-conjugate (Sigma) 1:5000 in PBS-T. Signal was developed with TMB and halted with stop solution, before reading at 450 nm.

NGF bioactivity and in vitro inhibition with antibodies was determined by analysing neurite outgrowth in response to NGF-mediated differentiation of rat adrenal phaeochromocytoma cells (PC-12). Type-I collagen (ThermoFisher Scientific, UK) (10 µg/ml) coated 24-well tissue culture plates were seeded in duplicate with ~7,500 PC-12 cells/well in assay media comprising RPMI 1640, GlutaMAX, HEPES (ThermoFisher Scientific, UK) further supplemented with 2% FBS, and penicillin/ streptomycin (1%), Sodium pyruvate (1%), non-essential amino acids (NEA) (1%), and incubated overnight at 37^o^C, 5% CO_2_. The following day media from wells were replaced with appropriate NGF-Ab mix in assay media prepared to give final concentrations of 10 ng/ ml NGF, with 0 – 100 µg/ml mAb (MAB256, R&D Systems) or purified IgG from vaccinated mice. NGF was omitted from negative control wells and antibodies were omitted from positive control wells. Cells were viewed after 4 days and up to 6 days. Brightfield images from several fields of view were captured on an inverted microscope Leica DM IL LED (Leica Microsystems (UK) Ltd), HI PLAN I 20x objective, using Q-Capture Pro 7software. Cells with and without neurite outgrowth (defined as extending cell body width) were counted and the proportion of neurite positive cells for each treatment were determined.

**Blood sampling:**

A sentinel cohort of 10 male C57BL6 mice was used alongside the main behavioural experiment to assess antibody titres throughout the experiment. Blood was sampled from the tail (lateral vein) in to blood collection tubes (Microvette CB300 Z, Sarstedt) and allowed to clot at room temperature for at least 1 h. The serum fraction was isolated by centrifugation at 10,000 x g for 3 min at room temperature and stored frozen at -20°C until further use in ELISA (described above).

**Surgically induced OA mouse model.**

**Animals:** All surgeries were performed following the procedures approved by the UK Home Office (Animals Scientific Procedures Act 1986) and the guidelines issued by the International Association for the Study of Pain were adhered to. Mice were kept in approved animal-care facilities and were housed 5 per cage in standard individually ventilated cages, maintained with a 12 h/12h light/dark cycle at an ambient temperature of 21°C. Animals were fed a certified mouse diet (RM3 from Special Dietary Systems, Essex, UK) and water ad libitum.

**Surgical joint destabilization:** 10 week old male C57BL6 mice (Charles River, UK) were randomised to undergo surgical destabilisation by partial meniscectomy or sham surgery of the knee joint as previously described[24]. Briefly, animals were placed under general anaesthesia by inhalation of Isoflurane (Vetpharma, Leeds, UK) 3% induction, 1.5-2% maintenance in 1.5-2 L/min O2, and hindlimbs were shaved and prepared for aseptic surgery. 0.3 mg/ml buprenorphine (Vetergesic Alstoe Animal Health, UK) was administered subcutaneously to all animals for analgesic purposes. For the sham operation, the knee joint was exposed with the meniscotibial ligament identified and the incision was closed with sutures. For the PMX surgery, the meniscotibial ligament was transected as well as approximately 1mm of the medial meniscus removed.

**Weight bearing.**

Static weight bearing measurements were performed using the Linton Incapacitance Tester (Linton Instrumentation, Norfolk, UK), as previously described[8]. Briefly, mice were first acclimatised to the chamber during training sessions on two separate occasions over two weeks before experimental measurements. Mice were manoeuvred inside the chamber to stand with one hindlimb on each scale. The weight placed on each hindlimb was measured over a one second interval for at least three consecutive measurements. Results are expressed as a percentage of the weight placed on the operated limb versus the weight placed on the contralateral control limb. One female observer (I.v.L.) performed the measurements and was blinded to the treatment status of the mice until the end of behavioural tests.

**Histology**

At the conclusion of the behavioural experiment, mice were sacrificed and the ipsi- and contralateral knee joints were collected for histological analyses by sharp division at the proximal femur and distal tibia. The skin and surrounding tissue were removed and subsequently fixed in 10% formalin for 24 hours before decalcification in 20% formic acid for one week. The tissue was subsequently paraffin-embedded and coronal sections of 4 micron thickness were cut using 80 micron intervals. Tissue slides were stained using Safranin-O and FastGreen for microscopic inspection and histological scoring. Histological analyses of the knees were done by blinded graded observations from two observers following the Osteoarthritis Research Society International (OARSI) scoring system[25]. Each joint consisted of eight to twelve scored sections. Each quadrant surface (lateral femoral condyle and tibial plateau and medial femoral condyle and tibial plateau) within the joint was scored separately, the highest summed score of a single section was chosen as the mid-point and summed scores of the three adjacent sections (all being 80microns apart) either side, were taken to yield an average total across 7 sections for each joint, labelled ‘Averaged OARSI Score’.

**Statistical analyses**

Data are expressed as mean ± standard error of the mean (SEM) and were analysed using GraphPad Prism (GraphPad Software, San Diego, CA). In behavioural experiments of three groups, a repeated measures ANOVA with a Tukey multiple comparisons test was applied. In behavioural experiments of only two groups, a repeated measures ANOVA with a Bonferroni multiple comparisons test was applied. We meta-analysed the effect sizes of the analgesic response between mock and vaccinated animals at the time points of highest titre in the sentinel groups. Effect size estimates and standard errors were taken from the repeated measures ANOVA, and the fixed-effect meta-analysis was carried out using the R package “meta”, which also produced the forest plot. In gene expression data involving two independent groups, multiple t-tests with Bonferroni post-hoc for multiple comparisons was applied. In the case of three groups (histology and gene expression data) a ordinary two-way ANOVA with Tukey was applied to correct for multiple comparisons within one timepoint. In order to compare across time points within a group, an ordinary two-way ANOVA with a Bonferroni post hoc was applied.

**References in Methods:**

23 Aricescu AR, Lu W, Jones EY. A time- and cost-efficient system for high-level protein production in mammalian cells. *Acta Crystallogr D Biol Crystallogr* 2006;62:1243–50. doi:10.1107/S0907444906029799

24 Knights CB, Gentry C, Bevan S. Partial medial meniscectomy produces osteoarthritis pain-related behaviour in female C57BL/6 mice. *Pain* 2012;153:281–92. doi:10.1016/j.pain.2011.09.007

25 Glasson SS, Chambers MG, van den Berg WB, *et al.* The OARSI histopathology initiative - recommendations for histological assessments of osteoarthritis in the mouse. *Osteoarthritis and Cartilage* 2010;18:S17–S23. doi:10.1016/j.joca.2010.05.025

**Contributions**

A.E.T., I.v.L. and T.V. designed the studies. M.F.B. originated the concept of the vaccine. J.Z. and I.v.L. conducted the mouse surgery and I.v.L. conducted the behavioural studies. A.Z. provided VLP constructs and developed purification strategies. A.E.T. produced and characterised the CuMVtt^NGF^ vaccine. A.E.T. performed the vaccinations and immunological assays. I.P. performed the histological preparation and in conjunction with I.v.L. conducted the histological analysis. L.J. conducted and approved of the statistical analysis. J.A. conducted the ELISA experiment.

**Supplementary information**

Supplementary Figures: Figure S1 – Figure S4

Online Methods

Reporting Summary = details of statistical parameters, software and code, data availability, study design (sample size, data exclusion, replication, randomisation, blinding), Materials & experimental systems.
