# Supplementary material for: Active immunisation targeting nerve growth factor attenuates chronic pain behaviour in murine osteoarthritis
Source: Ann Rheum Dis. 2019 Mar 12;78(5):672–5. doi: 10.1136/annrheumdis-2018-214489 (PMC6517802; doi:10.1136/annrheumdis-2018-214489)
Supplement: Supplementary data [file annrheumdis-2018-214489supp007.docx]

**Supplementary Figure 1: Pain behaviour and cartilage degradation following partial meniscectomy (PMX)**

**(a)** Weight bearing difference indicative of painful behaviour over the course of OA where 100% represents equal weight distributed across R and L limbs. Statistical significance shown by repeated measures two-way ANOVA with Tukey post-hoc for multiple comparisons. * for Naive vs PMX, + for Naive vs Sham, # for Sham vs PMX **p* < 0.05, **p < 0.01, ****p* < 0.001. Bars represent SEM. **(b)** Representative coronal knee joint sections of the operated knee after sham- or PMX surgery.  **(c)** Histological scoring of cartilage degradation of the knee joints after sham- or PMX surgery. Statistical significance shown within a time point by ordinary two-way ANOVA with Tukey post hoc for multiple comparisons. Statistical difference shown across time points within one group by ordinary two-way ANOVA with Bonferroni post hoc for multiple comparisons. **(d, e)** Whole joint gene expression of NGF after sham- or PMX surgery. **(d)** Gene expression of sham- and PMX-operated joints compared with naive joints for the post-operative phase. **(e)** Gene expression of PMX-operated joints compared to sham operated joints for each time point. Bars represent mean SEM. **p* < 0.05, **p < 0.01, ****p* < 0.001, *****p* < 0.0001 compared in **(d)** by ordinary two-way ANOVA with Tukey post hoc for multiple comparisons and in **(e)** compared with sham by two-tailed t-test with Bonferroni post hoc for multiple comparisons. Group sample sizes are naive = 6, sham = 10, PMX = 14 for **(a)** and otherwise represented as one animal per data point.

**Supplementary Fig. 2.** **Vaccine preparation and validation of recombinant His-tagged NGF**

 (**a**) Agarose gel analysis with SYBR safe showing increased RNA in the presence of a native 3’UTR (right hand lanes). (**b**) Coommassie stained SDS-PAGE of vaccine in quadruplicate. Vaccine is comprised of CuMVtt (~24.5 kDa monomer subunit) that forms dimers and multimers following derivatisation with SMPH (CuMVtt-SMPH), indicated by additional higher order bands. The derivatised particle is conjugated to the recombinant NGF (~14 kDa) via a terminal cysteine creating coupled intermediates indicated by asterisks (***** monomer-NGF conjugate, ~38 kDa; ****** dimer-NGF conjugate, ~64 kDa). **M**, Bio-Rad Precision Plus protein standards. SMPH – Succinimidyl 6-((beta-maleimidopropionamido) hexanoate). **(c,d)** Correct conformation of expressed recombinant (His-NGF) was confirmed by comparing it with commercially sourced NGF (2.5S subunit) purified from mouse submaxillary gland (Native-NGF) and its ability to bind **(c)** the neutralising monoclonal antibody (MAB256) by indirect ELISA or **(d)** the high affinity receptor (TrkA-d5-Fc hybrid). Control wells were without NGF (No NGF).

**Supplementary Fig. 3**. **Broader antibody responses by vaccine treatment.**

Serum from vaccinated, mock-vaccinated and vaccine-naive animals (all PMX-operated) at the end of the prophylactic study (week 18) were assessed for **(a)** titres of anti-CuMV antibodies **(b)** total IgG and IgM antibodies, and **(c) (d)** rheumatoid factor. Groups were compared in (a) and (d) by ordinary two-way ANOVA with Bonferroni post hoc for multiple comparisons and in (b) by ordinary two-way ANOVA with Tukey post hoc for multiple comparisons

**Supplementary Fig. 4.** **Neurite outgrowth inhibition**

Bioactivity of expressed recombinant NGF was confirmed by differentiation of rat adrenal phaeochromocytoma cells (PC-12) grown on collagen type-I coated plates. The cells were treated with NGF and the percentage of cells exhibiting neurite growth recorded after 4 days. **(a)** NGF neutralisation was assessed by pre-incubation of NGF with increasing concentrations of mAb (black bars) prior to addition onto cells, resulting in just under 40% reduction in neurite positive cells by day 4. **(b)** The mean difference in neurite positive cells was calculated by subtracting mAb treated values from mAb free control. **(c, d)** Representative morphology of PC-12 cells before **(c)** and after treatment with NGF **(d)**. n.s. - not significant, ***** *p* < 0.05, ****** *p* < 0.01, by Bonferroni multiple comparisons test.
